# ﻿Two new *Periconia* species (Pleosporales, Ascomycota) in China

**DOI:** 10.3897/mycokeys.125.173913

**Published:** 2025-11-26

**Authors:** Xing-Guo Tian, Jia-Jun Han, Sinang Hongsanan, Yong-Zhong Lu, Samantha C. Karunarathna, Dan-Feng Bao, Saowaluck Tibpromma

**Affiliations:** 1 School of Food and Pharmaceutical Engineering, Guizhou Institute of Technology, Guiyang, 550003, China School of Food and Pharmaceutical Engineering, Guizhou Institute of Technology Guiyang China; 2 Guizhou Key Laboratory of Agricultural Microbiology, Guizhou Academy of Agricultural Sciences, Guiyang, 550009, China Guizhou Key Laboratory of Agricultural Microbiology, Guizhou Academy of Agricultural Sciences Guiyang China; 3 Department of Entomology and Plant Pathology, Faculty of Agriculture, Chiang Mai University, Chiang Mai, 50200, Thailand Chiang Mai University Chiang Mai Thailand; 4 Shenzhen Key Laboratory of Microbial Genetic Engineering, College of Life Science and Oceanography, Shenzhen University, Shenzhen, 518060, China Shenzhen University Shenzhen China; 5 Center for Yunnan Plateau Biological Resources Protection and Utilization & Yunnan International Joint Laboratory of Fungal Sustainable Utilization in South and Southeast Asia, College of Biology and Food Engineering, Qujing Normal University, Qujing, 655099, China Qujing Normal University Qujing China; 6 Engineering and Research Center for Southwest Biopharmaceutical Resource of National Education Ministry of China, Guiyang, China Engineering and Research Center for Southwest Biopharmaceutical Resource of National Education Ministry of China Guiyang China

**Keywords:** 2 new species, biodiversity, fungal taxonomy, *Periconia guangxiense*, *Periconia xishuangbannaensis*

## Abstract

During an investigation of fungal diversity in Guangxi and Yunnan provinces, two interesting hyphomycetes were collected and isolated. Based on morphology, these hyphomycetes were similar to *Periconia*-like taxa. To confirm their classification, morphological characterization and multigene phylogenetic inference (using LSU, ITS, SSU, and *tef*1-α) were employed. Based on phylogenetic analyses, our two collections clustered within *Periconia* (Periconiaceae) as distinct two taxa, and morphological analyses also confirmed that they are new species of *Periconia*. *Periconia guangxiense***sp. nov.** formed a well-supported monophyletic lineage (92% ML/1.00 PP) sister to *P. variicolor*, but morphologically, it is distinguishable by its branched, micronematous to semi-macronematous conidiophores. While *Periconia xishuangbannaensis***sp. nov.** formed a distinct lineage closely related to *P. guangxiense*; it is distinguished by its macronematous conidiophores and dark brown conidia. This study highlights the role of unique habitats in driving fungal diversity and contributes to the taxonomic refinement of the *Periconia* genus.

## ﻿Introduction

Periconiaceae was established by Nannizzi (1934) with *Periconia* as the type genus. Historically, Periconiaceae has long been neglected, and *Periconia* was included in Massarinaceae (Zhang et al. 2009; Hyde et al. 2013). Periconiaceae was formally established and placed in Pleosporales (Dothideomycetes) by Tanaka et al. (2015) based on phylogenetic analyses. Periconiaceae includes four accepted genera: *Bambusistroma*, *Flavomyces*, *Noosia*, and *Periconia*, along with a single lineage of “*Sporidesmium tengii*” (Tanaka et al. 2015). In the phylogenetic analyses, *Bambusistroma*, *Flavomyces*, *Noosia*, and *Sporidesmium tengii* were present in the *Periconia* lineage (Tanaka et al. 2015; Liu et al. 2017). Recent taxonomic revisions have synonymized *Bambusistroma* and *Noosia* with *Periconia* (Tian et al. 2022; Yang et al. 2022), while *Flavomyces* remains a monotypic genus within the *Periconia* clade due to insufficient morphological characterization (Knapp et al. 2015; Tian et al. 2022).

*Periconia* was first described by Tode (1791) with *P. lichenoides* as the type species. Phylogenetic analyses by Tanaka et al. (2015) confirmed its placement in Periconiaceae, distinct from Massarinaceae. *Periconia* has 238 epithets listed in Index Fungorum (http://www.indexfungorum.org/; accessed August 20, 2025). Among these, 29 species have been transferred to other genera (Tian et al. 2022; Yang et al. 2022). Most *Periconia* species are hyphomycetous, with only five reported sexual morphs: *P. didymosporum*, *P. homothallica*, *P. igniaria*, *P. prolifica*, and *P. pseudodigitata* (Tanaka et al. 2015; Su et al. 2023; Liao et al. 2024). Asexual morphs are characterized by macronematous to micronematous, branched or unbranched conidiophores with spherical apices, monoblastic or polyblastic conidiogenous cells, and catenate, spherical to subspherical, verruculose conidia (Liao et al. 2024; He et al. 2025). Sexual morphs feature globose, scattered or grouped, ascomata with 8-spored, fissitunicate or cylindrical asci, and hyaline, 1-septate, fusiform, smooth ascospores with an entire sheath (Tanaka et al. 2015).

*Periconia* species are globally distributed, predominantly in temperate and tropical regions (Numata et al. 1997; Kolomiets et al. 2008; Sarkar et al. 2019; Gunasekaran et al. 2021; Madagammana et al. 2024; He et al. 2025). They occupy diverse ecological roles as saprobes, endophytes, plant pathogens (e.g., *P. igniaria* causing leaf spots on yellow starthistle; Kolomiets et al. 2008), and rarely human pathogens (e.g., *P. keratitis* causing mycotic keratitis; Gunasekaran et al. 2021). Aquatic species, on the other hand, such as *P. aquatica* and *P. submersa* are less common (Dong et al. 2020; Shen et al. 2024; Hongsanan et al. 2025).

In this study, two fungal taxa were collected and isolated from submerged decaying wood in a river and on dead leaves of *Zea mays* in Guangxi and Yunnan provinces. Multilocus phylogenetic analyses of combined LSU, ITS, SSU, and *tef*1-α sequence data placed them in *Periconia*. This study describes two new taxa, *Periconia guangxiense* and *P. xishuangbannaensis*, with detailed descriptions and illustrations. The research enhances our understanding of the ecological roles that *Periconia* species play in their respective environments and supports ongoing efforts to map the extensive fungal diversity present in unique habitats in China.

## ﻿Materials and methods

### ﻿Sample collection, isolation, and morphological examination

Specimens were collected from submerged decaying wood in a Guangxi freshwater stream and dead *Zea mays* leaves in Guangxi and Yunnan provinces, China (Tian et al. 2024). Samples were tagged with the collection information (Rathnayaka et al. 2025), put in sterile zip-lock bags, and taken to the mycology laboratory for processing. Single-spore isolation and morphological observations followed the methods of Senanayake et al. (2020). Morphological characteristics were examined using a stereomicroscope (Motic SMZ-171, Wetzlar, Germany). Microstructures were photographed with a Nikon DS-Ri2 camera mounted on a Nikon ECLIPSE Ni-U microscope (New York, USA). Measurements were taken using Image Framework v.0.9.0.7, and images processed with Adobe Photoshop CS6 (Adobe Systems, San Jose, CA, USA) (Tian et al. 2024).

Specimens were deposited in the herbarium of Guizhou Academy of Agriculture Sciences (GZAAS), Guiyang, China, while living cultures were deposited in the Guizhou Culture Collection (GZCC), Guiyang, China. The Index Fungorum (IF) number for the new species was obtained from Index Fungorum (2025). The new taxon was established according to the guidelines outlined by Jeewon (2016) and Chethana et al. (2021).

### ﻿DNA extraction, PCR amplification, and sequencing

Genomic DNA was extracted from 2-week-old pure cultures using a BioFlux BSC14S1 kit (Hangzhou, China) following the manufacturer’s instructions. The genomic DNA was stored at 4 °C and subjected to polymerase chain reaction (PCR) to amplify partial gene regions using the following primers: the partial large subunit nuclear rDNA (LSU) with primer pairs LR0R/LR5 (Vilgalys and Hester 1990), the internal transcribed spacer (ITS) gene was amplified with primer pairs ITS4/ITS5 (White et al. 1990), SSU gene was amplified using primers NS1 and NS4 (White et al. 1990).

The PCR amplifications were carried out using LSU, ITS, and SSU following the method described by Tian et al. (2024). The PCR thermal cycle programs for LSU, ITS, and SSU were as follows: an initialization step of 95 °C for 4 min, followed by 35 cycles of 94 °C for 40 s, an annealing step at 55 °C for 55 s, an elongation step at 72 °C for 50 min and a final extension step of 72 °C for 10 min. PCR products were checked on 1% agarose gels and sequenced using the same primers by Sangon Biotech Co., Kunming, China.

### ﻿Phylogenetic analyses

Forward and reverse sequences were assembled with SeqMan and subjected to BLASTn in NCBI (https://blast.ncbi.nlm.nih.gov/Blast.cgi) to identify the most similar taxa. Sequences of Periconiaceae were retrieved from GenBank (https://www.ncbi.nlm.nih.gov/) based on recent publications (Bao et al. 2025; He et al. 2025; Sun et al. 2025) and are listed in Table 1. Single gene sequence alignment was generated with the MAFFT v.7 online program (http://mafft.cbrc.jp/alignment/server/, 22 January 2025; Katoh and Standley 2013), trimmed using trimAl v 1.2 (Capella-Gutiérrez et al. 2009) and concatenated using Sequence Matrix. The FASTA alignment formats were changed to PHYLIP (for RAxML) and NEXUS formats (for BI) by Aliview 2.11. Multigene phylogenetic analyses were conducted using maximum likelihood (ML) and Bayesian inference (BI).

**Table 1.** Taxon names, strain numbers, and GenBank accession numbers of the fungal taxa used in this study.

Species name	Strain/ voucher NO.	GenBank accession numbers			
		ITS	LSU	SSU	*tef*1-α
*Bambusistroma didymosporum*	MFLU 15-0058	KP761734	KP761731	KP761738	KP761728
***Flavomyces fulophazii***	**CBS 135761**	**NR_137960**	**NG_058131**	**NG_061191**	/
*Flavomyces fulophazii*	CBS 135664	KP184000	KP184039	KP184081	/
***Lentithecium aquaticum***	**CBS 123099**	**NR_160229**	**NG_064211**	**NG_016507**	**GU349068**
***Lentithecium clioninum***	**KT1149A**	**LC014566**	**AB807540**	**AB797250**	**AB808515**
*Lentithecium clioninum*	KT1220	LC014567	AB807541	AB797251	AB808516
***Massarina cisti***	**CBS 266.62**	/	**AB807539**	**AB797249**	**AB808514**
*Massarina eburnea*	CBS473.64	/	GU301840	GU296170	GU349040
*Morosphaeria ramunculicola*	KH220	/	AB807554	AB797264	AB808530
*Moroxphaeria velatispora*	KH221	LC014572	AB807556	AB797266	AB808532
***Periconia algeriana***	**CBS 321.79**	**MH861212**	**MH872979**	/	/
***Periconia alishanica***	**NCYU 19-0160**	**MW063165**	**MW063229**	/	**MW183790**
***Periconia ananasi***	**MFLUCC 21-0155**	**OL753685**	**OL606153**	**OL606142**	**OL912946**
***Periconia aquatica***	**MFLUCC 16-0912**	**KY794701**	**KY794705**	/	**KY814760**
***Periconia arecacearum***	**MFLU 19-0803**	**PP592462**	**PP621090**	**PP639222**	**PP828795**
***Periconia arecacearum***	**SNT19A**	**PP592462**	**PP621090**	**PP639222**	**PP828795**
***Periconia artemisiae***	**KUMCC 20-0265**	**MW448657**	**MW448571**	**MW448658**	**MW460898**
***Periconia atropurpurea***	**CBS 381.55**	**MH857524**	**MH869061**	/	/
***Periconia banksiae***	**CBS 129526**	/	**NG_064279**	/	/
*Periconia byssoides*	MFLUCC 18-1548	MK347794	MK348013	MK347902	MK360070
*Periconia byssoides*	MFLUCC 17-2292	MK347751	MK347968	MK347858	MK360069
*Periconia byssoides*	MAFF 243869	LC014582	AB807569	AB797279	AB808545
*Periconia byssoides*	MFLUCC 18-1553	MK347806	MK348025	MK347914	MK360068
*Periconia caespitosa*	TC-2018a	MH051906	MH051907	/	/
***Periconia calamagrostidicola***	**CBS150887**	**PP791432**	**PP791460**	/	**PP780620**
*Periconia celtidis*	MFLU 19-2784	MW063162	MW063226	/	/
***Periconia chengduensis***	**CGMCC 3.23930**	**OP955987**	**OP956012**	**OP956056**	**OP961453**
***Periconia chiangraiensis***	**MFLUCC 21-0164**	**OL753686**	**OL606154**	**OL606143**	**OL912947**
***Periconia chimonanthi***	**KUMCC 20-0266**	**MW448660**	**MW448572**	**MW448656**	**MW460897**
***Periconia circinata***	CBS 263.37	MW810265	MH867413	/	MW735660
***Periconia citlaltepetlensis***	**IOM 325319**	**MH890645**	**MT625978**	/	/
*Periconia cookei*	MFLUCC 17-1399	MG333490	MG333493	/	MG438279
*Periconia cookei*	MFLUCC 17-1679	/	MG333492	/	MG438278
*Periconia cortaderiae*	MFLUCC 15-0453	KX965733	KX954402	/	KY320574
***Periconia cortaderiae***	**MFLUCC 15-0457**	**KX965732**	**KX954401**	**KX986345**	**KY310703**
*Periconia cortaderiae*	MFLUCC 15-0451	KX965734	KX954403	KX986346	KY429208
*Periconia cynodontis*	CGMCC 3.23927	OP909925	OP909921	OP909920	OP961434
***Periconia cyperacearum***	**CPC 32138**	**NR_160357**	**NG_064549**	/	/
***Periconia delonicis***	**MFLUCC 17-2584**	/	**NG_068611**	**NG_065770**	**MK360071**
*Periconia digitata*	CBS510.77	LC014584	AB807561	AB797271	AB808537
***Periconia dujuanhuensis***	**KUNCC 23-13482**	**PQ340469**	**PP189907**	/	**PQ456957**
***Periconia elaeidis***	**MFLUCC 170087**	**MG742713**	**MH108552**	**MH108551**	/
***Periconia endophytica***	**ZHKUCC 23-0995**	**OR995582**	**OR995588**	**PP277722**	**PP025968**
*Periconia endophytica*	ZHKUCC 23-0996	OR995583	OR995589	PP277723	PP025969
***Periconia epilithographicola***	**CBS 144017**	**NR_157477**	/	/	/
***Periconia festucae***	**CGMCC 3.23929**	**OP955973**	**OP955998**	**OP956042**	**OP961439**
***Periconia floridana***	**CBS 150884**	**NR_197917**	**NG_244042**	/	/
***Periconia guangxiense***	**GZAAS 25-0735**	**PX118886**	**PX118882**	**PX118890**	**PX522310**
*Periconia guangxiense*	GZAAS 25-0733	PX118887	PX118883	PX118891	PX522311
***Periconia genistae***	**CBS 322.79**	**MH861213**	**MH872980**	/	/
***Periconia homothallica***	**KT916**	**AB809645**	**AB807565**	**AB797275**	/
***Periconia hongheensis***	**KUNCC 23-13550**	**PQ340467**	**PP189905**	/	**PQ456958**
*Periconia hydei*	ZHKUCC 24-2102	PV434172	PV436641	PV437587	PV588048
***Periconia hydei***	**ZHKUCC 24-2101**	**PV434171**	**PV436640**	**PV437586**	**PV588047**
*Periconia igniaria*	CBS 379.86	LC014585	AB807566	AB797276	AB808542
*Periconia igniaria*	CBS 845.96	LC014586	AB807567	AB797277	AB808543
***Periconia imperatae***	**CGMCC 3.23931**	**OP955984**	**OP956009**	**OP956053**	**OP961450**
***Periconia kunmingensis***	**KUMCC 18-0173**	**MH892346**	**MH892399**	**OR225814**	**MH908963**
*Periconia lateralis*	CBS 292.36	MH855804	MH867311	/	/
***Periconia linzhiensis***	**HKAS 144526**	**PQ684989**	**PQ675408**	**PQ675368**	**PQ671465**
*Periconia macrospinosa*	CBS 135663	KP183999	KP184038	KP184080	/
*Periconia macrospinosa*	REF144	JN859364	JN859484	/	/
*Periconia minutissima*	MFLUCC 15-0245	KY794703	KY794707	/	/
*Periconia minutissima*	MUT2887	MG813227	/	/	/
***Periconia motuoensis***	**KUNCC24-17924**	**PQ373177**	**PQ438054**	**PQ438116**	**PQ661220**
***Periconia muchuanensis***	**CGMCC 3.25599**	**PQ067868**	**PQ067698**	**PQ066546**	**PQ278553**
***Periconia neobrittanica***	**CPC 37903**	**NR_166344**	**NG_068342**	/	/
***Periconia neominutissima***	**CBS 149514**	**OQ628478**	**OQ629060**	/	**OQ627950**
***Periconia palmicola***	**MFLUCC 14-0400**	/	**NG_068917**	**MN648319**	**MN821070**
***Periconia penniseti***	**CGMCC 3.23928**	**OP955971**	**OP955996**	**OP956040**	**OP961437**
***Periconia philadelphiana***	**CBS 149681**	**OQ628486**	**OQ629068**	/	**OQ627953**
*Periconia prolifica*	DBOF129	JQ724490	/	/	/
*Periconia prolifica*	DBOF23	JQ724384	/	/	/
*Periconia prolifica*	DBOF74	JQ724435	/	/	/
*Periconia prolifica*	DBOF153	JQ724513	/	/	/
***Periconia prolifica***	**CBS 209.64T**	**MH858422**	**MH870050**	/	/
*Periconia pseudobyssoides*	MFLU 17-1970	MG333491	MG333494	/	MG438280
*Periconia pseudobyssoides*	MAFF 243868	LC014587	AB807568	AB797278	AB808544
*Periconia pseudobyssoides*	MAFF 243874	LC014588	AB807560	AB797270	AB808536
*Periconia pseudodigitata*	KT644	LC014589	AB807562	AB797272	AB808538
*Periconia pseudodigitata*	KT1195A	LC014590	AB807563	AB797273	AB808539
***Periconia sahariana***	**CBS 320.79**	**MW444854**	**MH872978**	/	/
***Periconia salina***	**MFLU 19-1235**	**MN047086**	**MN017846**	**MN017912**	/
*Periconia shannanensis*	HKAS 134943	PP968554	PP968557	PP968560	PQ226772
***Periconia sichuanensis***	**CGMCC 3.25598**	**PQ067867**	**PQ067697**	**PQ066545**	**PQ278551**
***Periconia spodiopogonis***	**CGMCC 3.23932**	**OP955963**	**OP955988**	**OP956032**	**OP961429**
***Periconia submersa***	**MFLUCC 16-1098**	**KY794702**	**KY794706**	/	**KY814761**
***Periconia thailandica***	**MFLUCC 17-0065**	**KY753887**	**KY753888**	**KY753889**	/
***Periconia thysanolaenae***	**KUMCC 20-0262**	**MW442967**	/	/	**MW460896**
*Periconia variicolor*	SACCR-64	DQ336713	/	/	/
*Periconia variicolor*	BHE4	OR269168	/	/	/
*Periconia variicolor*	isolate L5	OP881885	/	/	/
*Periconia variicolor*	isolate 39JAN	ON208681	/	/	/
*Periconia variicolor*	strain F5	MF170861	/	/	/
*Periconia verrucosa*	MFLUCC 17-2158	MT310617	MT214572	MT226686	MT394631
***Periconia wurfbainiae***	**ZHKUCC 23-0999**	**OR995586**	**OR995592**	**PP277726**	**PP025972**
*Periconia wurfbainiae*	ZHKUCC 23-1000	OR995587	OR995593	PP277727	PP025973
***Periconia xishuangbannaensis***	**GZAAS25-0707**	**PX118888**	**PX118884**	**PX118892**	**PX522312**
*Periconia xishuangbannaensis*	GZAAS25-0734	PX118889	PX118885	/	/
***Periconia yangjiangensis***	**ZHKUCC 23-0997**	**OR995584**	**OR995590**	**PP277724**	**PP025970**
*Periconia yangjiangensis*	ZHKUCC 23-0998	OR995585	OR995591	PP277725	PP025971
***Periconia yantingense***	**SICAUCC 23-0047**	**PP060663**	**PP057956**	**PP003824**	**PP061142**
***Periconia yunnanensis***	**KUNCC 23-14259**	**PQ340468**	**PP189906**	/	**PQ456960**

Notes: Newly-generated sequences are indicated in cells with light red shading. Type strains are indicated in black/bold with light red shading. The symbol “/” indicates sequence not available.

Maximum likelihood analysis was done by the online RAxML-HPC v.8 on XSEDE Teragrid on CIPRES Science Gateway V. 3.3 (https://www.phylo.org, 22 August 2025) using the GTRGAMMA substitution model with 1,000 bootstrap replicates (Stamatakis et al. 2008; Miller et al. 2010; Dissanayake et al. 2020). The final tree was selected from suboptimal trees from each run by comparing likelihood scores.

Bayesian inference analysis was performed using MrBayes v. 3.2 on the XSEDE tool on the CIPRES portal (Miller et al. 2010). The models were selected as GTR+I+G for LSU, SSU, ITS, and *tef*1-α gene regions based on the best-fit model for BI analysis, which was estimated using MrModeltest v. 2.2 (Nylander 2004). Posterior probabilities (PP) (Rannala and Yang 1996; Zhaxybayeva and Gogarten 2002) were defined by the Bayesian Markov chain Monte Carlo (BMCMC) sampling method in MrBayes v.3.0b4 (Rannala and Yang 1996; Huelsenbeck and Ronquist 2001; Zhaxybayeva and Gogarten 2002). Two parallel runs were conducted using the default settings; six simultaneous Markov chains were run for 5,000,000 generations, and trees were sampled every 500^th^ generation. Phylogenetic trees were visualized using FigTree v1.4.4 (Rambaut 2012), and layouts were created with Adobe Illustrator CS5 v. 16.0.0 and Adobe Photoshop 2021 software (Adobe Systems, California, USA). All newly generated sequences were deposited in GenBank (https://www.ncbi.nlm.nih.gov/).

### ﻿Genealogical concordance phylogenetic species recognition (GCPSR) analysis

The genealogical concordance phylogenetic species recognition analysis is a model test used to check significant recombinant events (Tian et al. 2024). The data were analyzed using SplitsTree V4 with the pairwise homoplasy index (PHI) test to estimate recombination levels among closely related species (Bruen et al. 2006). A multilocus concatenated dataset with closely related species was used for the analysis. The relationships between closely related taxa were visualized by constructing split graphs using the LogDet transformation and splits decomposition options. PHI results below a 0.05 threshold (Φw < 0.05) indicate significant recombination in the dataset.

## ﻿Results

### ﻿Phylogenetic analyses

**Figure 1.**
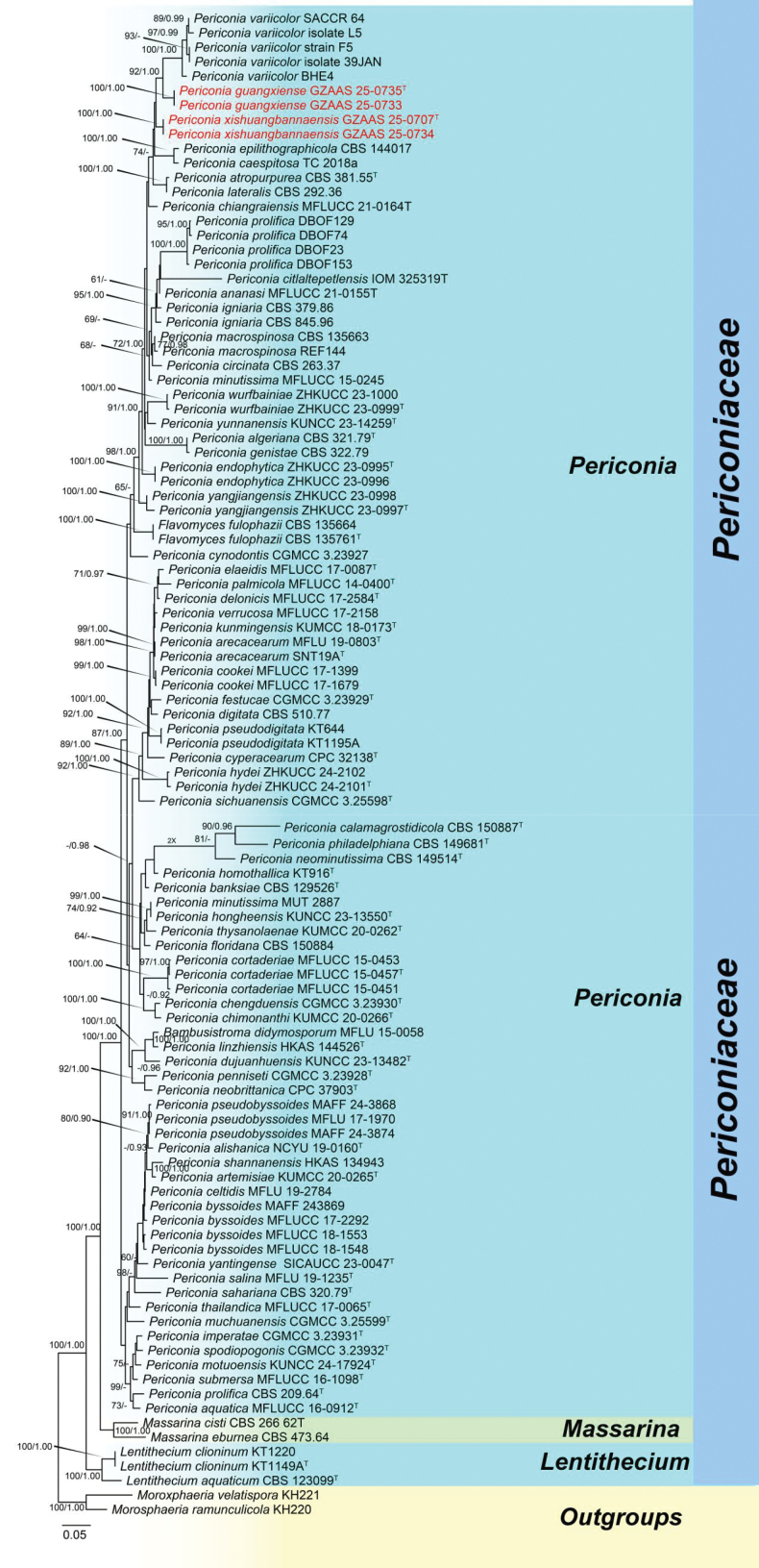
Phylogram generated from maximum likelihood (ML) analysis based on a concatenated alignment of ITS, LSU, SSU, and *tef*1-α sequences in Periconiaceae. Related sequences were obtained from Bao et al. (2025). The tree was rooted with *Morosphaeria ramunculicola* (KH220) and *M. velatispora* (KH221). Bootstrap support values for ML equal to or greater than 60% and clade credibility values greater than 0.90 from Bayesian inference analysis are labelled at each node. The new isolates are indicated in red, while “^T^” indicates holotype or ex-type strains.

The combined LSU, ITS, SSU, and *tef*1-α dataset comprised 104 strains, including four newly sequenced strains, with *Morosphaeria ramunculicola* (KH220) and *M. velatispora* (KH221) as the outgroup taxa. Multiple genes were concatenated, comprising 3,458 nucleotide characters, including gaps in the ITS (1–568), LSU (569–1,481), SSU (1,482–2,508), and *tef*1-α (2,509–3,438) regions. The RAxML analysis of the combined dataset yielded the best-scoring tree (Fig. 1) with a final ML optimization likelihood value of -25425.074632. The matrix contained 1,486 distinct alignment patterns, with 30.78% of the characters being undetermined or gaps. Estimated base frequencies were as follows: A = 0.237622, C = 0.257752, G = 0.267613, T = 0.237014; substitution rates: AC = 1.661890, AG = 2.814269, AT = 1.647163, CG = 1.275088, CT = 9.578081, GT = 1.000000; gamma distribution shape parameter *α* = 0.265472.

In this study, our phylogenetic analyses yielded a topology similar to those reported in previous studies (Yang et al. 2022; Liao et al. 2024), and our isolates were placed within *Periconia* (Fig. 1). The new isolates (GZAAS 25-0733 and GZAAS 25-0735) formed an independent lineage basal to *Periconia variicolor* with 92% ML/1.00 PP bootstrap support, while the other new isolates (GZAAS 25-0707 and GZAAS 25-0734) formed a well-supported independent lineage within the genus with low statistical support. Additionally, other species of *Periconia* were well separated, whereas *Flavomyces fulophazii* clustered within *Periconia*, an observation similar to other trees (Knapp et al. 2015; Tian et al. 2022).

### ﻿Taxonomy

#### ﻿ Periconia guangxiense

Taxon classificationFungiPleosporalesPericoniaceae

X.G. Tian & D.F. Bao sp. nov.

91B757BB-5410-5275-830F-12D1E8E5639D

Index Fungorum number: IF904184

Fig. 2

##### Holotype.

GZAAS 25-0735. The holotype GZAAS 25-0735 is the representative specimen of *Periconia guangxiense*, collected from decaying submerged wood in a freshwater stream in Longlin City, Guangxi Province, China, on 24 November 2024, by Dan-Feng Bao (collection code: NPJ 8-30/B199). This holotype specimen is deposited in the herbarium of Guizhou Academy of Agricultural Sciences (GZAAS) in Guiyang, China, while its corresponding ex-type living culture (GZCC 25-0705) is stored in the Guizhou Culture Collection (GZCC).

##### Etymology.

Named after the type location, “Guangxi”.

##### Description.

***Saprobic*** on decaying submerged wood in freshwater habitats. ***Sexual morph*** Undetermined. ***Asexual morph*** Hyphomycetous. ***Colonies*** effuse on natural substrate, scattered, hairy, dark brown to dark. ***Mycelium*** composed of cottony hyphae, forming dark clusters with conidia. ***Conidiophores*** micronematous to semi-macronematous, mononematous, branched, caespitose, straight or flexuous, hyaline when young, turning brown to dark brown, cylindrical, septate, smooth-walled. ***Conidiogenous cells*** 19–28 × 2.5–4.5 μm (x̄ = 23 × 3.5 μm, n = 6), mono- to polyblastic, determinate, discrete on stipe, intercalary, smooth to minutely verruculose, ellipsoid to cylindrical, brown. ***Conidia*** 6–7.5 μm diam. (x̄ = 6.8 μm, n = 60), subglobose to globose, aseptate, reddish-brown to dark brown, arising at one or more points on the curved surface of the conidiogenous cell, in branched chains, bud scars or disjunctors present at the site of attachment, verruculose.

**Figure 2.**
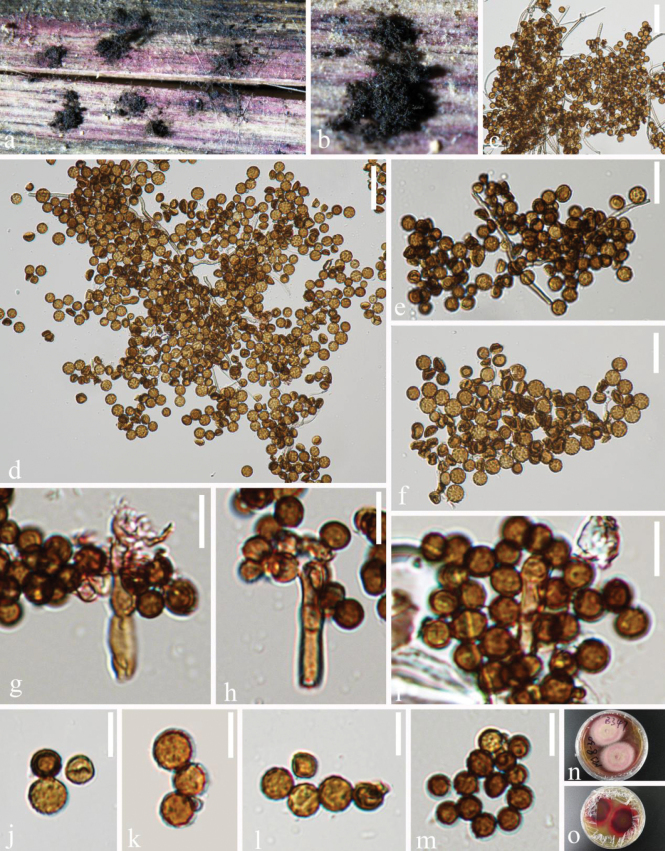
*Periconia guangxiense* (GZAAS25-0735, holotype). **a, b.** Colonies on submerged decaying wood; **c–f.** Conidial masses; **g–i.** Conidiogenous cells with conidia; **j–m.** Conidia; **n–o.** Culture on PDA from obverse and reverse view. Scale bars: 30 μm (**c–d**); 20 μm(**e–f**); 10 μm (**g–m**).

##### Culture characteristics.

Conidia germinated on PDA within 24 hr, reaching 20 mm diam. in 2 weeks at 25 °C. Colonies on PDA with white mycelia on the surface, cottony, circular, and flattened; reverse reddish brown with a pink margin, not sporulate on PDA after two weeks.

##### Material examined.

China • Guangxi Province, Longlin City, on decaying submerged wood, 24 Nov. 2024, D.F. Bao, NPJ 8-30 (B199) (GZAAS 25-0735, holotype), ex-type, GZCC 25-0705; *ibid.* NPJ 8-12 (GZAAS 25-0733, isotype), ex-isotype, GZCC 25-0704.

##### GenBank numbers.

GZCC 25-0705: ITS = PX118886, LSU = PX118882, SSU = PX118890, tef1-α = PX522310; GZCC 25-0704: ITS = PX118887, LSU = PX118883, SSU = PX118891, tef1-α = PX522311.

##### Notes.

In the phylogenetic analyses of the combined ITS, LSU, SSU, and *tef-1α* sequence data, our newly obtained strains of *Periconia guangxiense* (GZAAS 25-0735) formed a distinct lineage and clustered sister to *P. variicolor* strains (SACCR 64, isolate 5, F5, isolate 39JAN, BHE4) with 92% ML and 1.00 PP statistical supports (Fig. 1). Morphologically, *Periconia guangxiense* (GZAAS25-0735, holotype) can be easily distinguished from *P. variicolor* (CBS 120374) by micronematous, branched conidiophores, while *P. variicolor* has macronematous and unbranched conidiophores. *Periconia guangxiense* also has a smaller size range of conidia (6–7.5 µm vs. 7.5–9.5 μm) than the holotype of *P. variicolor* (CBS 120374) (Cantrell et al. 2007). In addition, pairwise nucleotide comparisons showed that ITS of *Periconia guangxiense* (GZCC 25-0705) differed 40/508 bp (7.87%, with gaps) from that of *P. variicolor* (SACCR-64), which strongly supports our strains as a new species. The PHI test revealed no significant recombination event between our strains (GZAAS25-0735 and GZAAS25-0733) and the closely related taxa. The significant recombination between our two strains indicates that they are conspecific (Φw = 0.98) (Fig. 4). Therefore, *Periconia guangxiense* is introduced as a distinct new species of *Periconia*.

#### ﻿ Periconia xishuangbannaensis

Taxon classificationFungiPleosporales

X.G. Tian & D.F. Bao sp. nov.

628B5F8C-D9B3-5D2C-B2A0-1309D03794B0

Index Fungorum number: IF904185

Fig. 3

##### Holotype.

GZAAS 25-0707. The holotype GZAAS 25-0707 is the representative specimen of *Periconia xishuangbannaensis*. It was collected from dead leaves of *Zea mays* in Xishuangbanna City, Yunnan Province, China, on September 17, 2021, by Xing-Guo Tian (collection code: corn3-2). This holotype specimen is deposited in the herbarium of Guizhou Academy of Agricultural Sciences (GZAAS) in Guiyang, China, while its corresponding ex-type living culture (GZCC 25-0677) is stored in the Guizhou Culture Collection (GZCC).

##### Etymology.

Named after the type location, “Xishuangbanna”.

##### Description.

***Saprobic*** on dead leaves of corn. ***Sexual morph*** Undetermined. ***Asexual morph*** Hyphomycetous. ***Colonies*** effuse on the natural substrate, gregarious, dark brown to black, floccose, and spore masses spread on the host surface. ***Mycelium*** partly superficial, composed of septate, brown hyphae. ***Conidiophores*** 229–247 × 4.5–7 μm (x̄ = 243 × 5.5 µm, n = 5), macronematous, mononematous, caespitose, erect, cylindrical, straight or slightly flexuous, branched, septate, cylindrical, smooth, brown, smooth-walled. ***Conidiogenous cells*** 6.5–14 × 4–6 μm (x̄ = 10 × 5 μm, n = 15), holoblastic, often polyblastic, terminal or intercalary, integrated, discrete on stipe, subcylindrical, smooth to verruculose, pale brown to brown. ***Conidia*** 4.5–7 μm (x̄ = 6 μm, n = 30) diam., globose, aseptate, pale brown to brown, becoming dark brown at maturity, arising at one or more points on the curved surface of the conidiogenous cell, catenate, minutely verruculose to short-spinulose.

**Figure 3.**
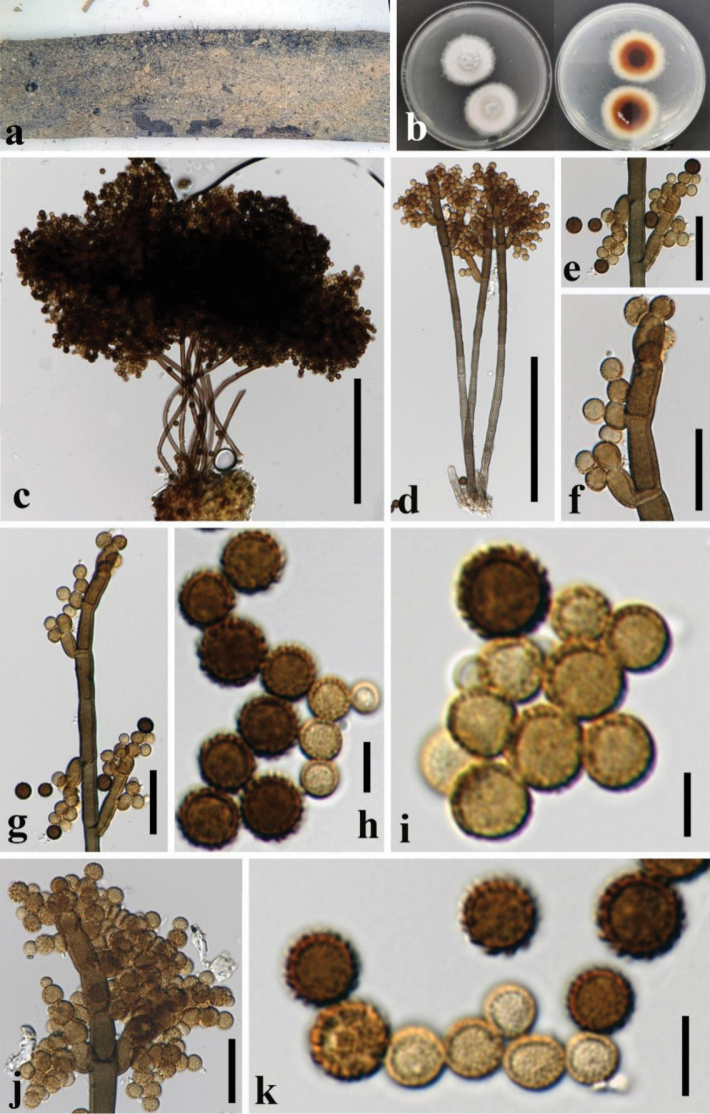
*Periconia xishuangbannaensis* (GZAAS 25-0707, holotype). **a.** Colonies on natural substrate; **b.** Colonies on PDA from above and below; **c**–**d.** Conidiophores, conidiogenous cells, and conidia; **e**–**g, j.** Conidiogenous cells and conidia; **h**, **i, k.** Conidia. Scale bars: 100 μm (**c–d**), 20 μm (**e–g, j**), 5 μm (**h, i, k**).

##### Culture characteristics.

Conidia germinated on PDA within 24 hr, reaching 15 mm diam. in 2 weeks at 25 °C. Colonies on PDA with white mycelia on the surface, cottony, circular, and flattened; reverse of the colony is brown with a white margin, not sporulate in PDA after two weeks.

##### Material examined.

China • Yunnan Province, Xishuangbanna City, on dead leaves of *Zea mays*, 17 Sep. 2021, X.G. Tian, corn3-2 (GZAAS 25-0707 holotype) , ex-type, GZCC 25-0677; *ibid.* corn3-13 (GZAAS 25-0734, isotype), ex-isotype, GZCC 25-0703.

##### GenBank numbers.

GZCC 25-0677: ITS = PX118888, LSU = PX118884, SSU = PX118892, tef1-α = PX522312; GZCC 25-0703: ITS = PX118889, LSU = PX118885.

##### Notes.

**Figure 4.**
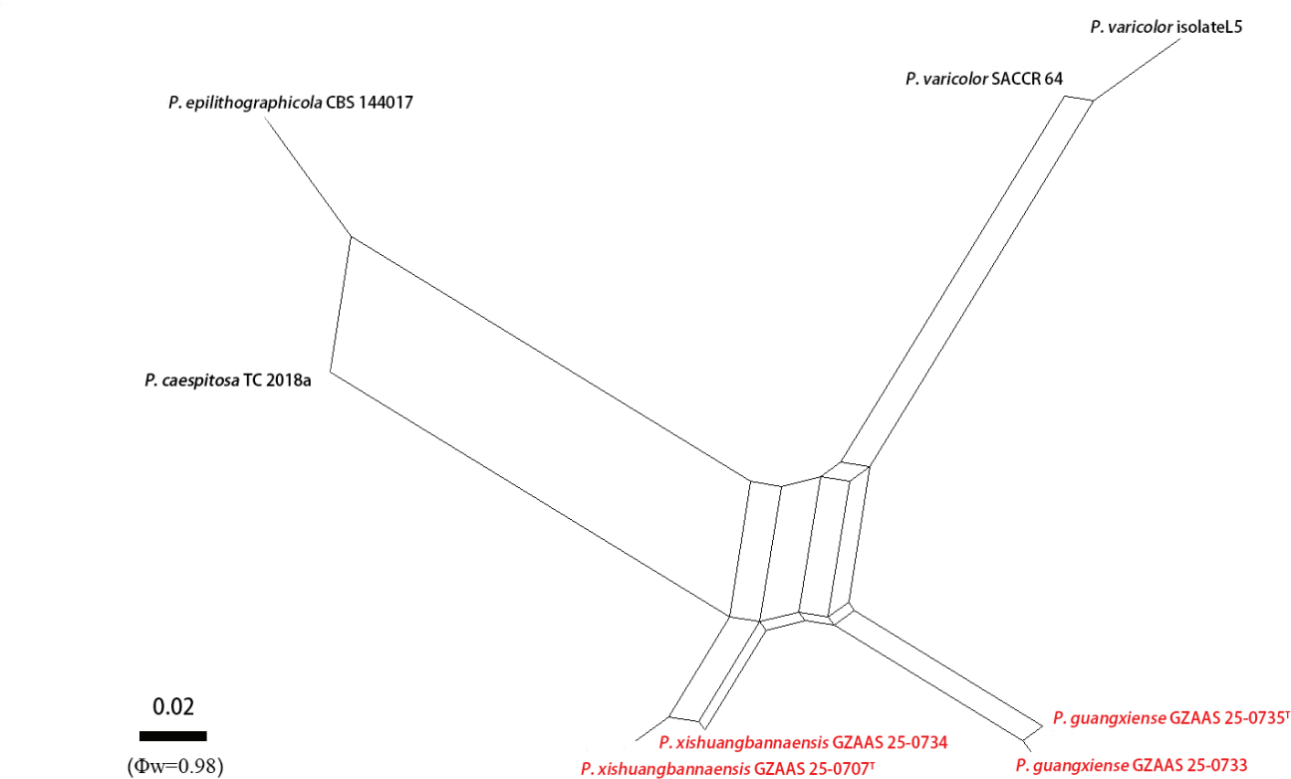
Results of the PHI test of the new species and closely related species using both LogDet transformation and splits’ decomposition. The new strains are in red font, and “^T^” indicates holotype or ex-type strains.

In the phylogenetic analyses of the combined ITS, LSU, SSU, and *tef*1-*α* sequence data, our newly obtained strains of *Periconia xishuangbannaensis* formed a distinct lineage and clustered as a sister clade to *P. variicolor* (SACCR-64) and *P. guangxiense* without statistical support (Fig. 1). Morphologically, *P. xishuangbannaensis* can be easily distinguished from *P. variicolor* by macronematous, branched conidiophores, while *P. variicolor* has macronematous and micronematous, unbranched conidiophores. *Periconia xishuangbannaensis* also has a smaller size range of conidia (4.5–7 µm vs. 7.5–9.5 μm) than *P. variicolor* (Cantrell et al. 2007). In addition, pairwise nucleotide comparisons showed that ITS of *Periconia xishuangbannaensis* (GZCC 25-0677) was significantly different from that of *P. variicolor* (SACCR-64) (43/540 bp, 7.96%, with gaps) and *P. guangxiense* (GZAAS 25-0735) (38/508 bp, 7.5%, with gaps). The PHI test revealed no significant recombination event between our strains (GZCC 25-0677 and GZCC 25-0703) and the closely related taxa. The significant recombination between our two strains indicates that they are conspecific (Φw = 0.98) (Fig. 4). Therefore, *Periconia xishuangbannaensis* is introduced as a new species.

## ﻿Discussion and conclusion

In this study, we introduce two new saprobic *Periconia* species, underscoring the role of unique habitats in shaping fungal diversity and advancing the taxonomic delimitation of the genus *Periconia*. Both new species lack sexual morphs, a trait consistent with most *Periconia* taxa, in which asexual states predominate. The conidiogenesis and verruculose conidia of *P. guangxiense* and *P. xishuangbannaensis* align with the generic characteristics of *Periconia* (Ellis 1971), while their conidiophores exhibit significant differences: micronematous and branched in *P. guangxiense*, versus macronematous and unbranched in *P. xishuangbannaensis*, serving as diagnostic features for species-level differentiation. The 7.8% ITS sequence divergence between *P. guangxiense* and its sister species *P. variicolor* exceeds the typical threshold for fungal species delimitation (Jeewon 2016).

*Periconia* species are predominantly terrestrial, functioning as saprobes or plant pathogens, with only a few documented aquatic taxa (e.g., *P. aquatica*, *P. dujuanhuensis*, *P. hongheensis*, *P. hydei*, *P. submersa*, and *P. yunnanensis*) (Dong et al. 2020; Shen et al. 2024; Hongsanan et al. 2025). The discovery of *P. guangxiense* in freshwater habitats expands the known ecological range of the genus and suggests that fungal diversity in freshwater ecosystems remains underexplored.

*Periconia xishuangbannaensis* formed an independent lineage in our phylogenetic analyses, but this placement received no statistical support (Fig. 1). This uncertainty underscores the potential impact of expanded taxon sampling and the inclusion of additional protein-coding markers. These measures can significantly improve phylogenetic resolution and stabilize the topological structure within Periconiaceae. Future studies, with their focus on multigene approaches incorporating more conserved loci, hold the promise of better resolving interspecific relationships within this morphologically diverse genus, inspiring further research in the field.

After 2020, there has been a notable increase in new *Periconia* species discoveries across various provinces/regions in China based on both morphology and phylogenetic analyses. In Sichuan, eight new species of *Periconia* were identified (*P. penniseti*, *P. spodiopogonis*, *P. imperatae*, *P. sichuanensis*, *P. chengduensis*, *P. cynodontis*, *P. festucae*, and *P. muchuanensis*) (Xu et al. 2022; Su et al. 2023; Yu et al. 2024); in Guizhou, *P dicranopteridis* was introduced by Bao et al. (2025); a rich haul of new taxa, including *P. artemisiae*, *P. chimonanthi*, *P. hydei*, *P. kunmingensis*, *P. pseudobyssoides*, *P. yantingensis*, and *P. yunnanensis* were introduced from Yunnan (Yang et al. 2022; Phookamsak et al. 2024; Shen et al. 2024; Wang et al. 2024; Hongsanan et al. 2025); in Taiwan, *P. alishanica* and *P. celtidis* were reported (Tennakoon et al. 2021); in Xizang, *P. motuoensis*, *P. shannanensis*, and *P. linzhiensis* were discovered (He et al. 2025; Sun et al. 2025); and in Guangdong, *P. endophytica*, *P. wurfbainiae*, and *P. yangjiangensis* were identified (Liao et al. 2024). These discoveries collectively demonstrate that *Periconia* exhibits remarkable species diversity and a wide distribution across China. Together with the 24 new species described worldwide between 2020 and 2025, they underscore both the genus’s underexplored diversity and the critical need for continued taxonomic and ecological studies.

## Supplementary Material

XML Treatment for Periconia guangxiense

XML Treatment for Periconia xishuangbannaensis

## ﻿Additional information

### Use of AI

No use of AI was reported.

### Author ORCIDs

Xing-Guo Tian https://orcid.org/0000-0002-8124-1281

Jia-Jun Han https://orcid.org/0009-0000-4919-369X

Sinang Hongsanan https://orcid.org/0000-0003-0550-3152

Yong-Zhong Lu https://orcid.org/0000-0002-1033-5782

Samantha C. Karunarathna https://orcid.org/0000-0001-7080-0781

Dan-Feng Bao https://orcid.org/0000-0002-5697-4280

Saowaluck Tibpromma https://orcid.org/0000-0002-4706-6547

### Data availability

All of the data that support the findings of this study are available in the main text.
